# G protein coupled estrogen receptor attenuates mechanical stress-mediated apoptosis of chondrocyte in osteoarthritis via suppression of Piezo1

**DOI:** 10.1186/s10020-021-00360-w

**Published:** 2021-08-28

**Authors:** Yi Sun, Ping Leng, Pengcheng Guo, Huanshen Gao, Yikai Liu, Chenkai Li, Zhenghui Li, Haining Zhang

**Affiliations:** 1 Department of Joint Surgery, The Affiliated Hospital of Qingdao University, Qingdao, 266000 China; 2 Department of Pharmacy, The Affiliated Hospital of Qingdao University, Qingdao, 266000 China; 3 Department of Joint Orthopedics, Weifang Hospital of Traditional Chinese Medicine, Weifang, 261000 China

**Keywords:** G protein coupled estrogen receptor, Osteoarthritis, Mechanical stress, Chondrocyte, Piezo1

## Abstract

**Background:**

Apoptosis of chondrocyte is involved in osteoarthritis (OA) pathogenesis, and mechanical stress plays a key role in this process by activation of Piezo1. However, the negative regulation of signal conduction mediated by mechanical stress is still unclear. Here, we elucidate that the critical role of G protein coupled estrogen receptor (GPER) in the regulation of mechanical stress-mediated signal transduction and chondrocyte apoptosis.

**Methods:**

The gene expression profile was detected by gene chip upon silencing Piezo1. The expression of GPER in cartilage tissue taken from the clinical patients was detected by RT-PCR and Western blot as well as immunohistochemistry, and the correlation between GPER expression and OA was also investigated. The chondrocytes exposed to mechanical stress were treated with estrogen, G-1, G15, GPER-siRNA and YAP (Yes-associated protein)-siRNA. The cell viability of chondrocytes was measured. The expression of polymerized actin and Piezo1 as well as the subcellular localization of YAP was observed under laser confocal microscope. Western blot confirmed the changes of YAP/ Rho GTPase activating protein 29 (ARHGAP29) /RhoA/LIMK /Cofilin pathway. The knee specimens of osteoarthritis model were stained with safranin and green. OARSI score was used to evaluate the joint lesions. The expressions of GPER and YAP were detected by immunochemistry.

**Results:**

Expression profiles of Piezo1- silenced chondrocytes showed that GPER expression was significantly upregulated. Moreover, GPER was negatively correlated with cartilage degeneration during OA pathogenesis. In addition, we uncovered that GPER directly targeted YAP and broadly restrained mechanical stress-triggered actin polymerization. Mechanism studies revealed that GPER inhibited mechanical stress-mediated RhoA/LIMK/cofilin pathway, as well as the actin polymerization, by promoting expression of YAP and ARHGAP29, and the YAP nuclear localization, eventually causing the inhibition of Piezo1. YAP was obviously decreased in degenerated cartilage. Silencing YAP caused significantly increased actin polymerization and activation of Piezo1, and an increase of chondrocyte apoptosis. In addition, intra-articular injection of G-1 to OA rat effectively attenuated cartilage degeneration.

**Conclusion:**

We propose a novel regulatory mechanism underlying mechanical stress-mediated apoptosis of chondrocyte and elucidate the potential application value of GPER as therapy targets for OA.

**Supplementary Information:**

The online version contains supplementary material available at 10.1186/s10020-021-00360-w.

## Introduction

Osteoarthritis (OA) is a chronic and degenerative disease of the joint, which is characterized by degeneration of cartilage, subchondral bone changes, and formation of osteophytes (Glyn-Jones et al. 2015). As chondrocytes are the only cells in articular cartilage, the decline of chondrocyte survival rate would result in the destruction of the articular cartilage (Geyer and Schonfeld 2018). In addition to genetic and environmental factors, mechanical stress has a crucial effect on the development of osteoarthritis by regulating chondrocyte apoptosis (Felson et al. 2000). Previous studies reported that mechanical stress upregulated the expression of Piezo1, a novel mechanical sensitive ion channel, in chondrocytes, which in turn increased the apoptosis of chondrocyte, and eventually led to cartilage degeneration (Li et al. 2017). Therefore, molecules possessing activation of Piezo1 induced by mechanical stress are urgently needed.

It was found that apoptosis of chondrocytes induced by mechanical stress was downregulated by cytochalasin D (cytoD), implying that cytoskeleton protein, especially the actin, may have vital roles in mechanical signal transduction during OA. In addition, chondrocytes from OA cartilage show actin polymerization, which renders OA cartilage, particularly susceptible to mechanical stress (Millward-Sadler et al. 2000). Meanwhile, OA cartilage activates signal pathway for contributing to actin polymerization compared to normal cartilage (Lambrecht et al. 2008). Thus, targeting actin dynamics signaling may be an effective approach to treat OA.

Signal transduction pathways of mechanical stress have been extensively studied (Zhu et al. 2016; Jorgensen et al. 2017; Lee et al. 2017a). Collectively, upon mechanical stress, membranes undergo structural changes facilitates recruitment of cytosolic factors (Halder et al. 2012). In response to mechanical stress, the subcellular localization of Yes-associated protein (YAP), a transcription coactivator, is transferred from nucleus to cytoplasm (Panciera et al. 2017; Chang et al. 2018). Cytoplasmic YAP downregulates the expression of Rho GTPase activating protein 29 (ARHGAP29) which known as the regulator of the RhoA/LIMK/cofilin pathway (Meng et al. 2018). Cofilin is an actin depolymerizing factor, which is blocked in function by activating this pathway (Qiao et al. 2017). Polymerized actin regulated by cofilin enhance the activation of Piezo1, which ultimately increase the apoptosis of chondrocyte (Li et al. 2017). However, the modulation of mechanical stress-mediated signaling pathways, particularly the regulation of upstream molecules, has not yet been elucidated.

Estrogen receptors include the conventional estrogen receptors (ERs) in the cytoplasm or nucleus, and the novel G protein coupled estrogen receptors (GPERs), which exert their regulatory functions by fast non genomic pathway and slow genomic pathway (Barton et al. 2018). Estrogen receptor regulate cell proliferation, apoptosis and metabolism, implying the essential role of estrogen receptor in maintaining intracellular homeostasis (Pakdel 2018). Previous studies illuminated the apoptosis of chondrocyte induced by mechanical stress significantly decreased upon estrogen treatment (Imgenberg et al. 2013). However, at present, mechanical stress associated-estrogen receptor type probably implicated in OA development still remains unclear.

Through this study, we investigated mechanical stress associated-estrogen receptor in chondrocytes and tried to discern estrogen receptor type closely related in mechanical stress-mediated signaling and explored their underlying effects on apoptosis of chondrocyte during OA process. Our research found that GPER was response to regulate mechanical stress transduction in chondrocyte. GPER suppresses mechanical stress-mediated RhoA/LIMK/cofilin activation, actin polymerization, and the activation of Piezo1 by promoting expression of YAP and ARHGAP29, as well as the YAP nuclear localization, consequently attenuates apoptosis of chondrocyte.

## Materials and methods

### Collection of articular cartilage

This study was approved by the medical ethics committee of the Affiliated Hospital of Qingdao University (QYFYKYLL-2017-06-12-05), and all patients written informed consent. Degenerative articular cartilage tissues from 15 females, aged 55–70 years (60.36 ± 10.44) years, were collected from patients undergoing total knee arthroplasty due to osteoarthritis. Control tissues were also collected from 10 females, aged 20–31 years (25.28 ± 5.37) years, who underwent surgery for fracture of tibial plateau. Specimens are separated into two parts. One part of the specimens was fixed in 4% paraformaldehyde for histological examination and analysis, the other part was immersed in PBS for isolation.

### Isolation and culture of OA chondrocytes

OA cartilage was washed three times with 100 μ/ml penicillin and 0.1 mg/ml streptomycin, and cut into 1 mm^3^ pieces. 0.25% trypsin (Hclone, Logan, USA) was added to the tissue pieces and digested at 37 °C for 30 min, and 0.2% type II collagenase (Roche diagnosis, Tokyo, Japan) at 37 °C for 4 h. After filtration with 70 μm nylon membrane, the suspension was centrifuged at 1200 rpm for 8 min. Cells were cultured in Dulbecco’s modified eagle’s medium/Ham’s F12 (DMEM/F-12; GIBCO, Waltham, USA) containing 15% FBS, (life technologies, Carlsbad, Canada), 100 μ/ml penicillin and 0.1 mg/ml streptomycin at 37 °C in a 5% CO_2_ incubator. The second-generation chondrocytes were taken for the follow-up experiment.

### RNA-mediated interference

siRNA for human Piezo1, GPER and YAP gene was obtained from Jima Gene Company (Shanghai, China). Chondrocytes were seeded in a six-well plate, and when chondrocytes reached 80% confluency, targeting siRNA or scrambled sequence wrapped by lentivirus with titer of 50 nM were added. Polybren (Sigma-Aldrich, St. Louis, USA) was used to facilitate the transfection process.

### Application of cyclic stress

Chondrocytes were digested with trypsin and cultured in 6-well collagen-coated BioFlex plates at a density of 2 × 10^6^. DMEM/F-12 containing 15% fetal bovine serum was added into each well. The serum-free DMEM/F-12 was used to replace the original culture medium when the cells grew to 80–90% confluence. After 24 h of culture, BioFlex plates were placed into the multi-channel stress loading system FX-4000T (Flexercell International, McKeesport, USA). The frequency was set to 6 cycles per minute with 20% surface elongation, and the cells were harvested after 24 h. Cells cultured under the same conditions without exposure to mechanical stress were used as the control group.

### Chip analysis

The total RNA was extracted by RNAiso Kit (Takara, Osaka, Japan). The total RNA samples were analyzed by Agilent 2100 for quality inspection. The amplified RNA (aRNA) was prepared by GeneChip 3′IVT Express Kit. After purification and fragmentation, aRNA was hybridized with the chip probe. Upon hybridization, the chip was washed and dyed, and then the image and original data were obtained by scanning. The screening criteria of the significant difference genes (SDGs) were: |fold change|> 2 and false discovery rate (FDR) < 0.05. Analysis of SDGs was carried out using the IPA Pathway Analysis, with samples being grouped by transfection condition, three replicates per group. Disease and function analysis were performed to categorize the considerably enriched functional classification in which SDGs operated.

### Detection of chondrocyte apoptosis

Annexin V-FITC/propidium iodide double staining was used to assess apoptosis and necrosis. According to the manufacturer's instructions of the Annexin V-FITC Apoptosis Detection Kit (R&D Systems, New Brighton, USA), samples were prepared for flow cytometric analysis. Viable cells were identified as Annexin-V−/PI−, early apoptotic cells as Annexin-V + /PI−, late apoptotic cells as Annexin-V + /PI +, and necrotic cells as Annexin-V−/PI +.

### Real-time polymerase chain reaction (RT-PCR)

The total RNA of chondrocytes is extracted as above mentioned. As per the manufacturer’s instructions which were provided with Prime Script RT reagent Kit (TaKaRa, Osaka, Japan), 1 μg RNA was reverse-transcribed into complementary DNA. The mRNA expression of GPER were detected by quantitative fluorescence Kit (TaKaRa, Osaka, Japan) on Roche Light Cycler 480 (R&D Systems, USA). The parameters of thermal cycle conditions included a 30 s pre-culture at 95 °C followed by 40 cycles at 95 °C for 5 s and 60 °C for 20 s. GAPDH as a housekeeping gene normalizes CT values. The relative gene expression was calculated by 2^−△△Ct^ method. Information about the sequence of the primers is provided in Table 1.

**Table 1:** Primer sequences

Target mRNA	Sequence (5′–3′)
GPER	Forward primer: 5′- TTAGGCCTGATACGTAGCTA -3′
	Reverse primer: 5′- AGGCATTTCGCGATTAAGTT -3′
GAPDH	Forward primer:5′- TGTGTCCGTCGTGGATCTGA -3′
	Reverse primer: 5′- TTGCTGTTGAAGTCGCAGGA -3′

### Western blot

Radio-immunoprecipitation assay buffer was added to chondrocyte for 30 min to extract total protein. The concentration of total protein was determined by BCA method, and separated by sodium dodecyl sulphate–polyacrylamide gel electrophoresis and transferred to the polyvinylidene difluoride membrane under constant current condition, and blocked with 5% skimmed milk at room temperature for 1.5 h. Rabbit anti-human BAX (Abcam, Cambridge, UK), Bcl-2 (Abcam, Cambridge, UK), GPER (Abcam, Cambridge, UK), YAP, ARHGAP29 (Abcam, Cambridge, UK), RhoA (Abcam, Cambridge, UK), active RhoA (Abcam, Cambridge, UK), LIMK, P-LIMK, cofilin, P-cofilin and GAPDH primary antibodies (dilution concentration 1:2000, Cell Signaling Technology, Boston, USA) were incubated overnight at 4 °C. Then goat anti-rabbit horseradish peroxidase-conjugated antibody (dilution concentration 1:2000, Cell Signaling Technology, Boston, USA) incubated at room temperature for 1 h. Target proteins were detected by enhanced chemiluminescence reagent. GAPDH was used as internal control.

### Immunofluorescence staining

OA chondrocytes were fixed with 4% paraformaldehyde for 30 min, then permeate with 0.03%Triton X-100 for 10 min. After washing with PBS, bovine serum albumin was used to block for 30 min, and YAP (Abcam, Cambridge, UK), Piezo1 (Novus, Colorado, USA) and Phalloidin (dilution concentration 1:500, Abcam, Cambridge, UK) were used for overnight at 4 °C. Then, they were incubated at room temperature for 1 h with Alexa labeled secondary antibody (Invitrogen, Carlsbad, USA). DAPI (Invitrogen, Carlsbad, USA) was used to dye for 10 min, and the image was taken by confocal microscope.

### OA model preparation

Eight-week-old SD rats (female, 200 g) were randomly divided into three groups: Sham + Negative control (NC), OA + NC and OA + G-1 group, 6 rats in each group. Surgical OA model was induced by resecting anterior cruciate ligament (ACL) and medial collateral ligament (MCL) on the right knee joint. Sham operation received the same incision without cutting off ACL and MCL. Rats in OA + G-1 group were intra-articular injected with G-1 (40 μg/kg/day, for 7 weeks) 1 week after the surgery. Rats in Sham + NC and OA + NC group were administrated with equal saline injections.

### Histological examination

Articular cartilage was fixed with 4% paraformaldehyde for 2 days, decalcified with 15% EDTA at pH 7.4 for about 8 weeks, dehydrated in graded ethanol solution, embedded in paraffin, and sectioned into 5 μm sections. Safranin O/fast green staining was performed on the knee joint specimens of rats. Cartilage was evaluated by two experienced pathologists in a blinded manner, and assessed by using an Osteoarthritis Research Society International (OARSI) scoring system as previous described (Kang 2020). The images were obtained using a light microscope (Olympus, Tokyo, Japan).

### Immunohistochemical staining

The decalcified sections were boiled in sodium citrate (pH 6.0) for 10 min to retrieve antigen, and incubated with 3% hydrogen peroxide for 10 min to block endogenous peroxidase activity. The non-specific binding was reduced by incubating the sections with 3% goat serum for 30 min at room temperature. Sections were incubated overnight at 4 °C, and the main antibodies were GPER (1:300, Abcam, Cambridge, UK), and YAP (1:300, Abcam, Cambridge, UK). The slides were visualized by a light microscope.

### Statistical analysis

SPSS 23.0 (IBM, Chicago, USA) was used for statistical analysis. All quantitative variables were expressed as mean ± standard deviation (SD). Student’s t test or one way analysis of variance (ANOVA) were used to compare differences between groups. *P* < 0.05 indicates that the difference has statistical significance.

## Results

### GPER is upregulated in chondrocytes exposed to mechanical stress after Piezo1 silencing

**Fig. 1:**
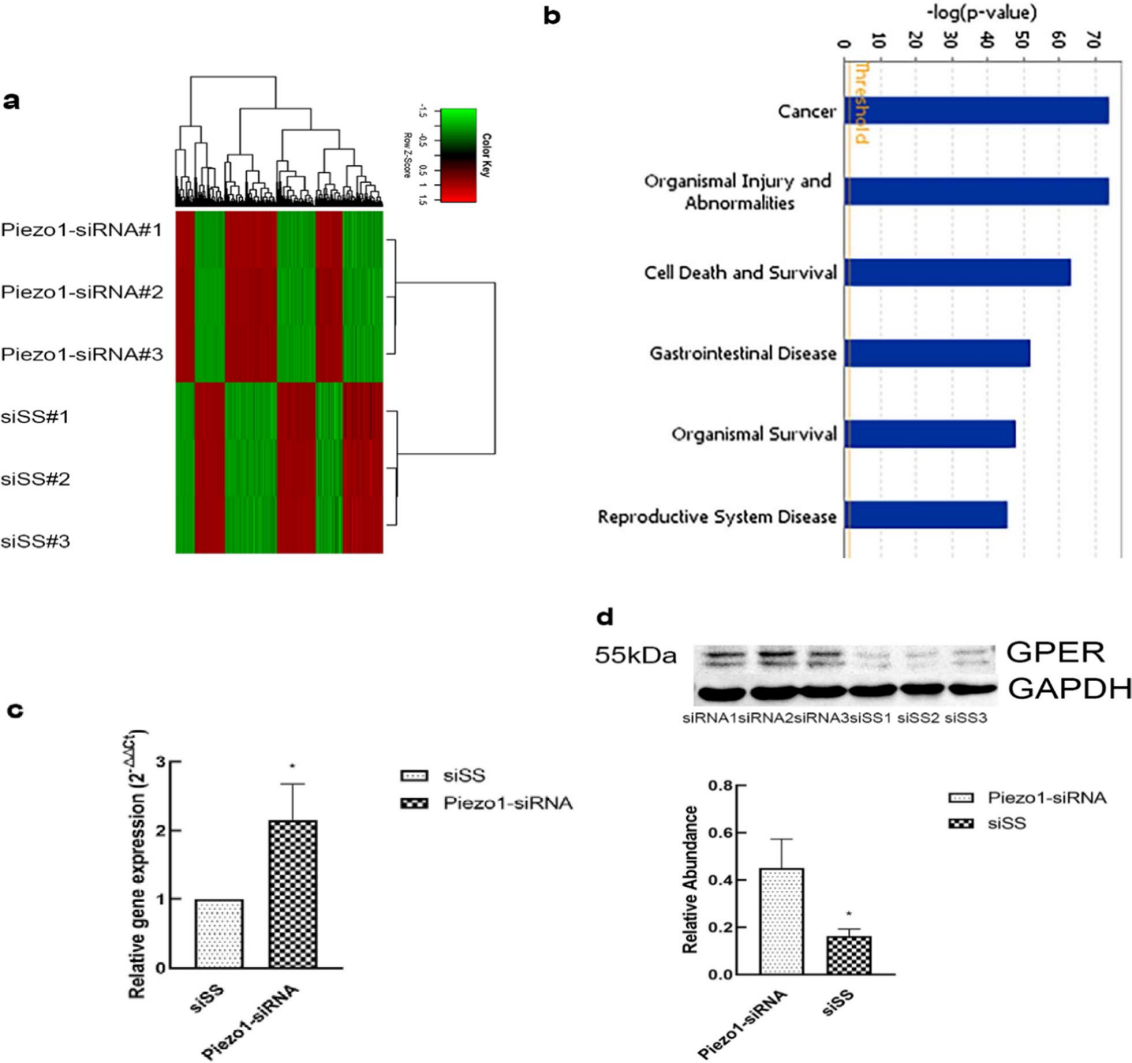
GPER is upregulated in chondrocytes exposed to mechanical stress after Piezo1 silencing. **a** Heatmap of differential gene expression values upon Piezo1 silencing in chondrocytes exposed to mechanical stress. **b** Disease and function analysis of differential gene expression values upon Piezo1 silencing in chondrocytes exposed to mechanical stress. **c** RT-qPCR analysis of GPER genes in Piezo1-silenced chondrocytes compared with non-silenced chondrocytes (n = 3). **d** Protein expression of GPER in Piezo1-silenced chondrocytes compared with non-silenced chondrocytes (n = 3). All data are presented in mean ± SD *P < 0.05, compared with non-silenced chondrocytes

In order to explore the molecular and regulatory networks that interact with Piezo1, we explored the effect of Piezo1-siRNA on the expression profile of chondrocytes exposed to mechanical stress. Silencing Piezo1 resulted in estrogen-associated transcriptional signature when significant gene expression differences are grouped and visualized as a Heatmap (Fig. 1a). Further studies showed that the novel estrogen receptor GPER was significantly up-regulated after Piezo1 silencing, while the conventional ERα and ERβ had no significant change. Disease and function analysis indicated that the differential genes were mainly involved in cell death and survival, suggesting that Piezo1 play a crucial role in chondrocyte survival (Fig. 1b). The mRNA expression measurement established that GPER were increased with transfection of Piezo1-siRNA (Fig. 1c). The Western blot results were consistent with the mRNA expression (Fig. 1d).

### GPER expression is decreased in human osteoarthritic cartilage

**Fig. 2:**
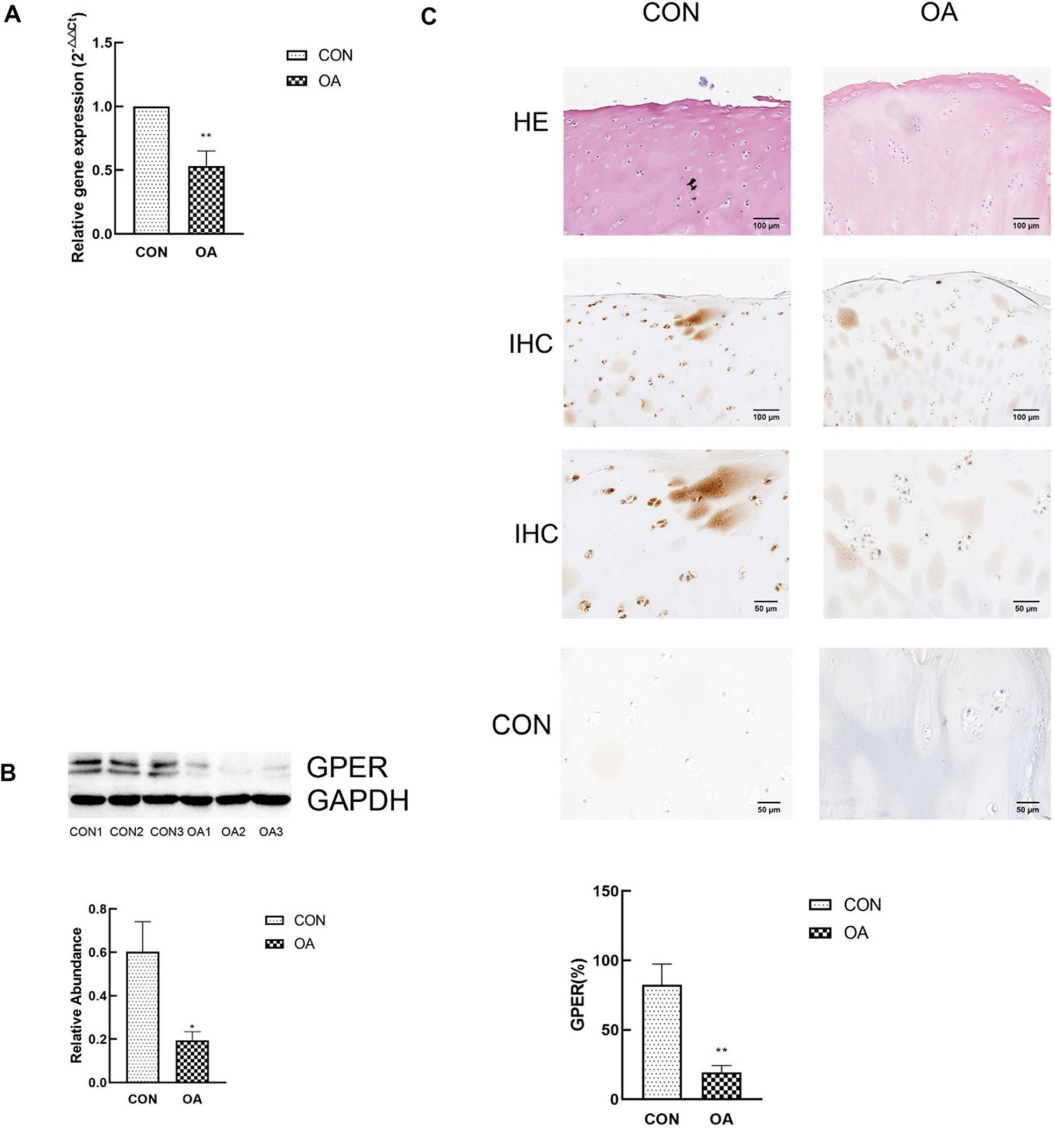
GPER expression is decreased in human osteoarthritis cartilage. **a** RT-qPCR analysis of GPER genes in degenerative cartilage compared with control cartilage (n = 10). **b** Protein expression of GPER in degenerative cartilage compared with control cartilage (n = 3). **c** Immunohistochemical staining of degenerative cartilage and control cartilage without (control) and with anti-GPER antibody (n = 10). All data are presented in mean ± SD *P < 0.05, compared with control cartilage. **P < 0.01, compared with control cartilage

In order to explore the effect of GPER on OA, we first detected the mRNA expression of GPER in the control joint tissues of 10 patients with tibial plateau fracture and 10 patients with knee osteoarthritis. Quantitative mRNA expression showed a significant reduction in GPER in osteoarthritis articular cartilage compared with the control group (Fig. 2a). In order to further confirm the relationship between GPER and osteoarthritis cartilage degeneration, we measured the protein level by Western blot (Fig. 2b), and got consistent results. GPER expression was also investigated in human cartilage. Immunohistochemical staining and corresponding quantification of GPER showed that GPER is significantly downregulated in degenerative cartilage compared with control cartilage (Fig. 2c).

### Activation of GPER reduces the apoptosis of chondrocytes induced by mechanical stress

**Fig. 3:**
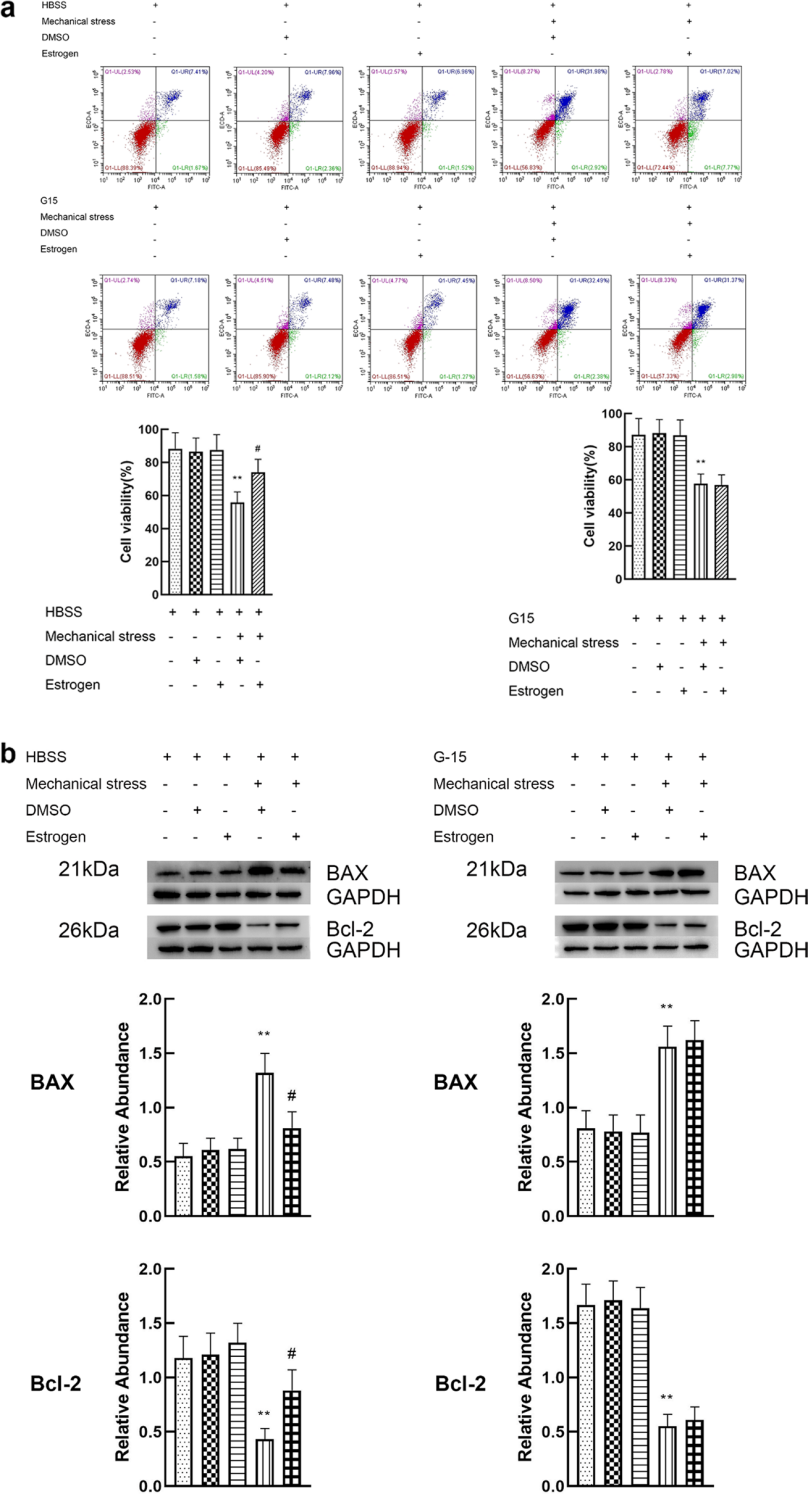

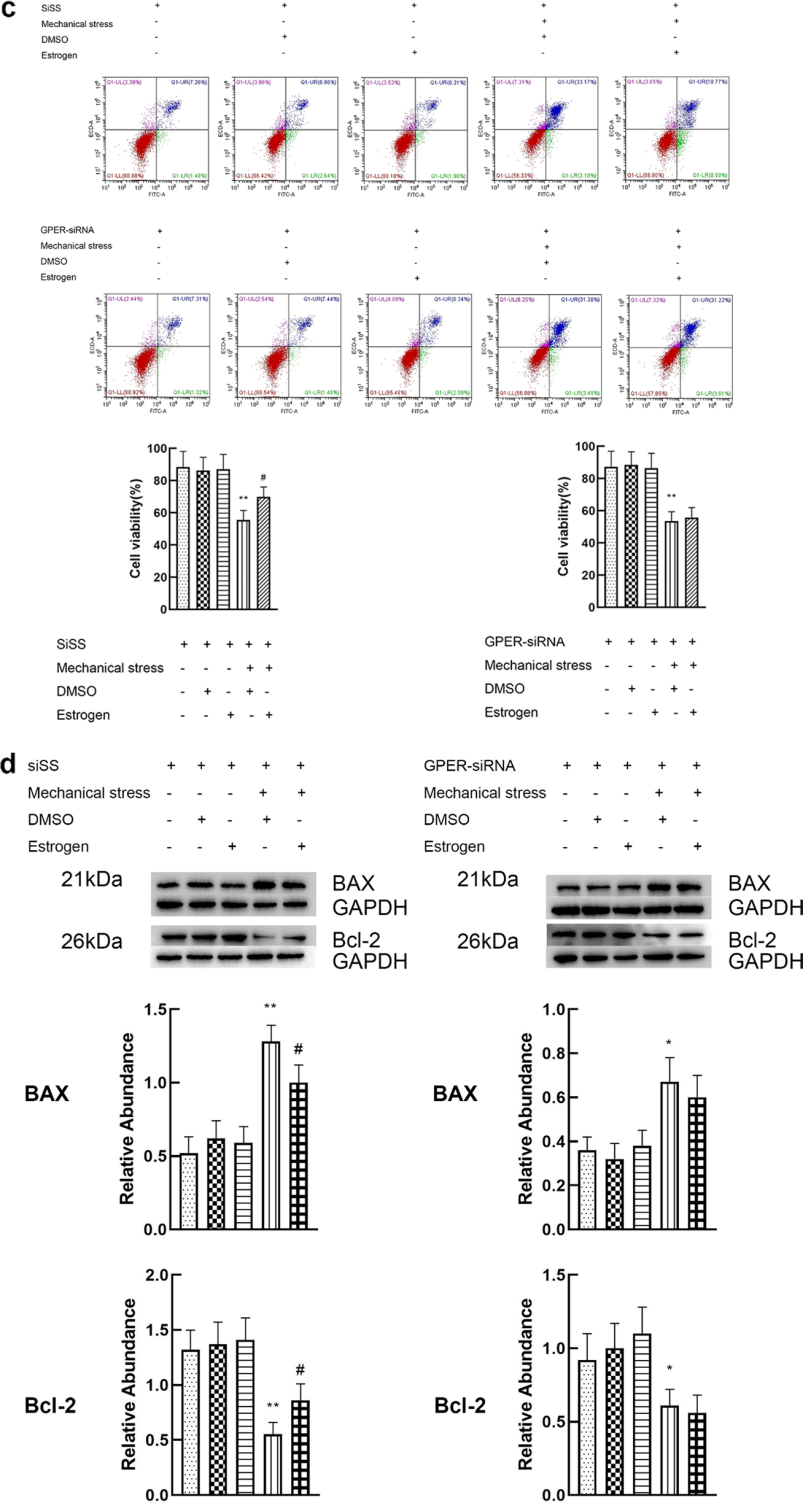

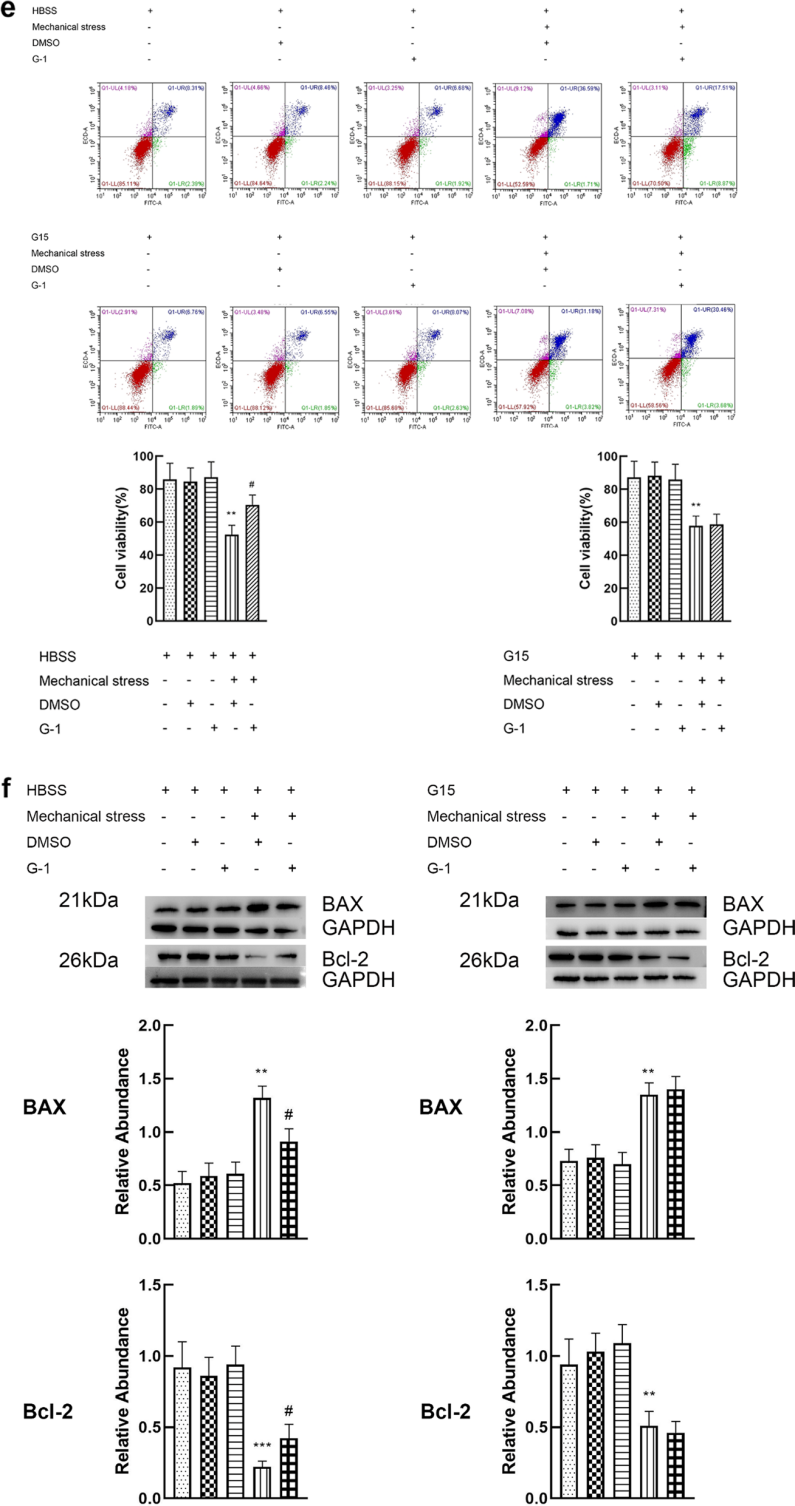

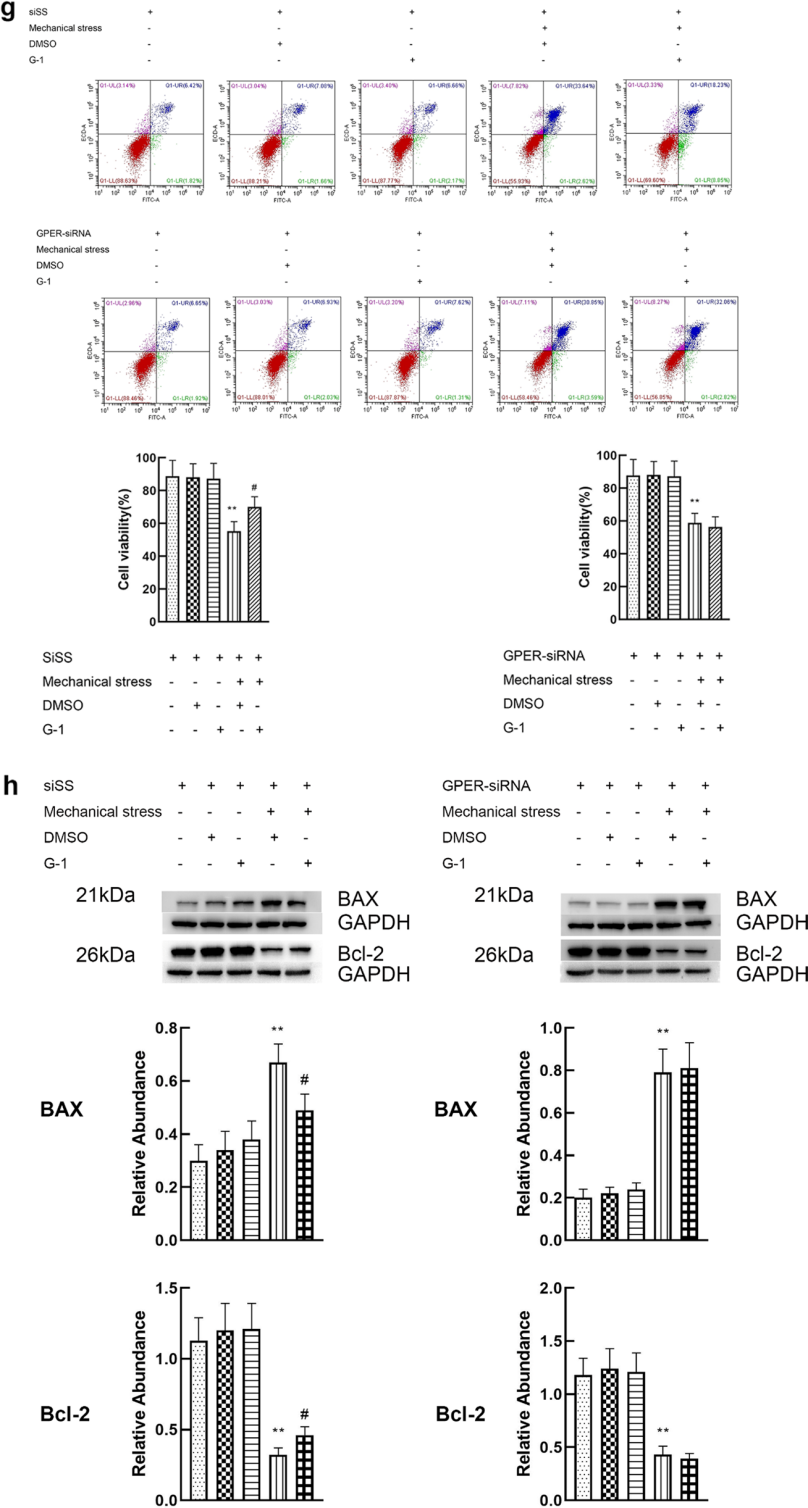
Activation of GPER reduces the apoptosis of chondrocytes induced by mechanical stress. **a** Cell viabilities for chondrocytes treated with mechanical stress, 8 μM estrogen, and 8 μM G15 (n = 3). **b** Protein expression of Bax and Bcl-2 for chondrocytes treated with mechanical stress, 8 μM estrogen, and 8 μM G15 (n = 3). **c** Cell viabilities for chondrocytes with GPER or scrambled siRNA after treatment with mechanical stress, and estrogen (n = 3). **d** Protein expression of Bax and Bcl-2 for chondrocytes with GPER or scrambled siRNA after treatment with mechanical stress, and estrogen (n = 3). **e** Cell viabilities for chondrocytes treated with mechanical stress, 10 μM G-1, and 8 μM G15. **f** Protein expression of Bax and Bcl-2 for chondrocytes treated with mechanical stress, 10 μM G-1, and 8 μM G15. **g** Cell viabilities for chondrocytes with GPER or scrambled siRNA after treatment with mechanical stress, and G-1 (n = 3). **h** Protein expression of Bax and Bcl-2 for chondrocytes with GPER or scrambled siRNA after treatment with mechanical stress, and G-1 (n = 3). All data are presented in mean ± SD (n = 3). *P < 0.05, compared with DMSO group. **P < 0.01, compared with DMSO group. ^#^P < 0.05, compared with mechanical stress + DMSO group. ^##^P < 0.01, compared with mechanical stress + DMSO group

The doses of G-1 and estrogen were 10 μM and 8 μM respectively. The dose–effect curves are shown in Additional file 1. Cell viability, and expression of apoptosis related proteins (Bcl-2 and Bax) were used to evaluate apoptosis of chondrocyte. Chondrocytes exposed to 24 h mechanical stress presented an increase in apoptosis, characterized by a significant decline in cell viability, increased expression of BAX and decreased expression of Bcl-2. When the chondrocytes exposed to 24 h mechanical stress were treated with 8 μM estrogen, a decrease in apoptosis (increase in cell viability, decreased expression of BAX and increased expression of Bcl-2) was found. However, estrogen alone did not play a significant role in viability and expression of BAX and Bcl-2 (Fig. 3a, b). G15, the inhibitor of GPER, attenuated the effect of estrogen on the inhibition of chondrocyte apoptosis induced by mechanical stress. These results suggest that GPER instead of ER mediates the antiapoptotic effect of estrogen. To further prove the effect of GPER on resisting apoptosis induced by mechanical stress, GPER was knocked down using siRNA. Compared with the scrambled siRNA, GPER-siRNA effectively blocked the anti-apoptosis effect of estrogen, which was consistent with the results of the application of inhibitors (Fig. 3c, d). Moreover, we explored the potential role of G-1 in mechanical stress-induced chondrocyte apoptosis. Treatment with G-1 alone did not cause a significant increase in apoptosis (Fig. 3e, f). The G-1-mechanical stress group had an increased viability, decreased expression of BAX and increased expression of Bcl-2 compared with mechanical stress group (Fig. 3e, f). To confirm if G-1 treats chondrocyte through GPER activation, GPER was inhibited using siRNA knockdown. G-1 treatment of chondrocyte transfected with scrambled siRNA had negligible change, whereas the GPER-siRNA treated cells showed a significant increase in apoptosis (Fig. 3g, h).

### GPER inhibits mechanical stress-mediated actin polymerization and Piezo1 activation

**Fig. 4:**
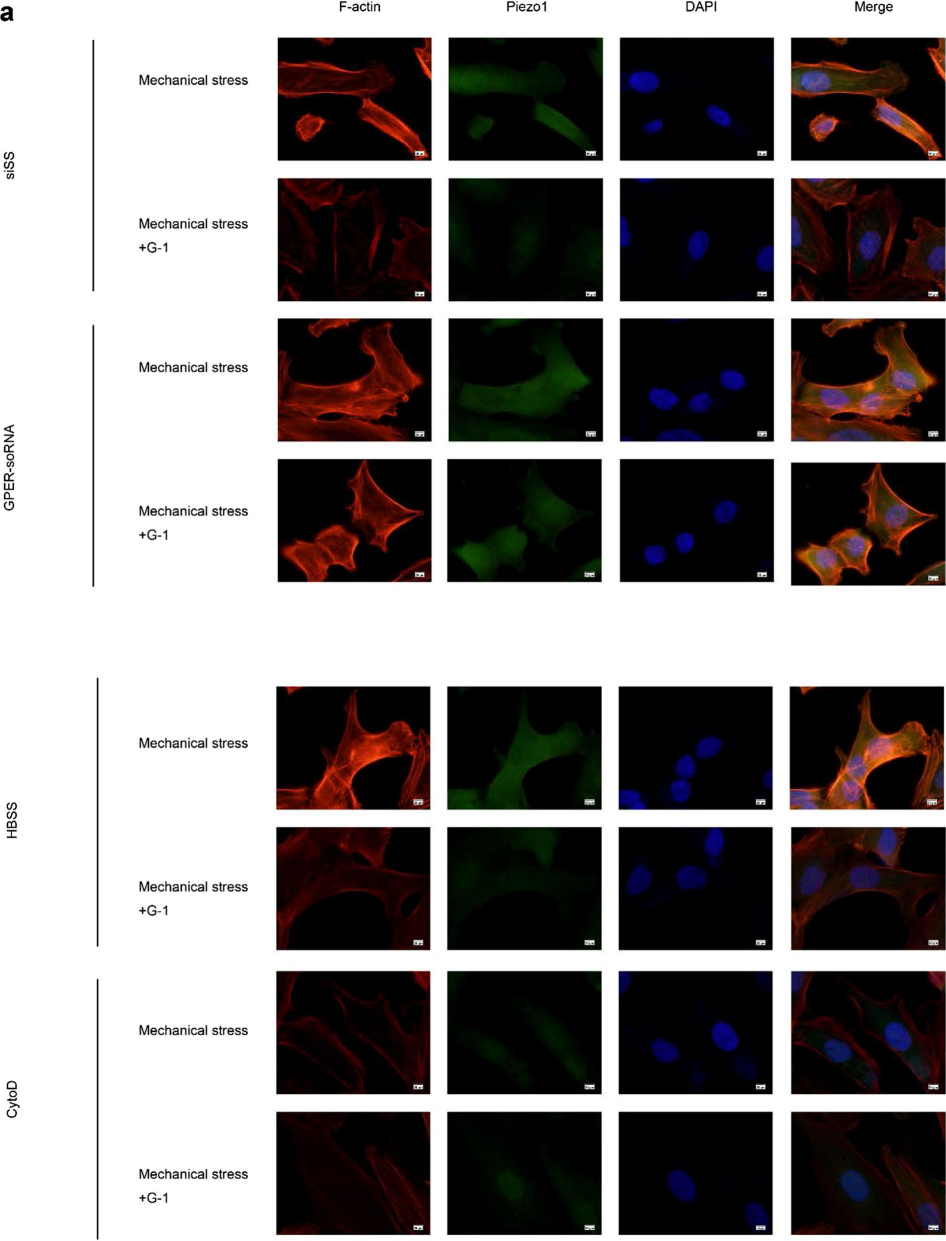

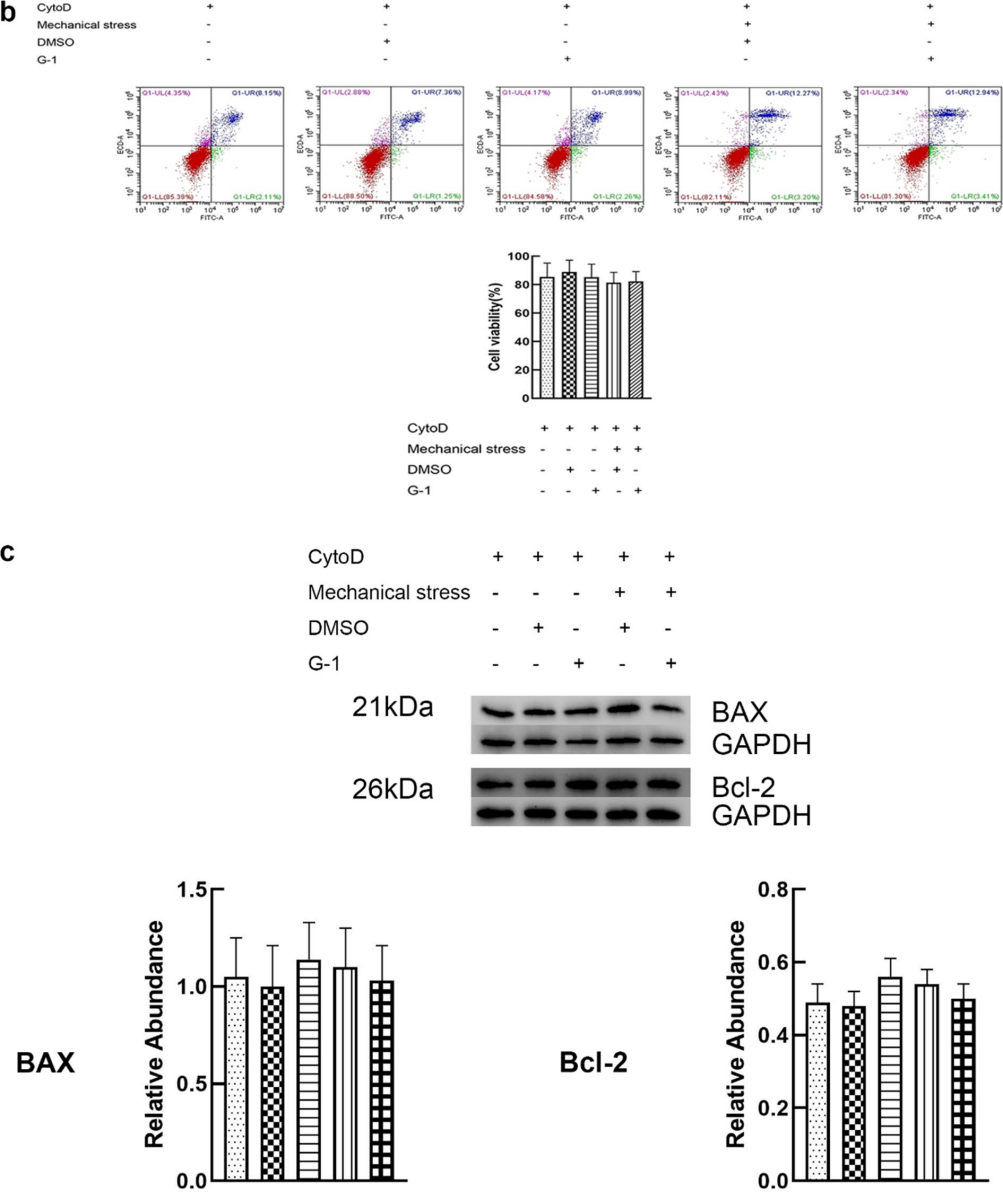
GPER inhibits mechanical stress-mediated actin polymerization and Piezo1 activation. **a** Immunofluorescence staining F-actin and Piezo1 (n = 3). **b** Cell viabilities for chondrocytes treated with mechanical stress, 10 μM G-1, and 8 μM cytoD (n = 3). **c** Protein expression of Bax and Bcl-2 for chondrocytes treated with mechanical stress, 10 μM G-1, and 8 μM cytoD (n = 3). All data are presented in mean ± SD

In order to uncover the role of GPER in mechanical stress-induced actin polymerization and Piezo1 activation, we treated chondrocytes with G-1 or mechanical stress. Compared with the control group, mechanical stress upregulated the fluorescence intensity of Piezo1 in chondrocytes. However, G-1 treatment could significantly attenuate the increased fluorescence intensity in this process. (Fig. 4a). Activation of Piezo1 is closely related to actin polymerization. To clarify the role of actin, chondrocyte was treated with actin inhibitor, CytoD. Compared with the mechanical stress group, the expression of Piezo1 in chondrocytes treated with CytoD decreased (Fig. 4a), the cell viability and expression of Bcl-2 increased as well as the expression of BAX decreased (Fig. 4b, c). G-1-mechanical stress treated chondrocyte had significantly decreased actin-fluorescence intensity compared with mechanical stress treated cells (Fig. 4a), suggesting that GPER inhibits mechanical stress-mediated activation of Piezo1 via actin depolymerization.

### GPER regulates actin polymerization via YAP-ARHGAP29-RhoA-LIMK-Cofilin Signaling

**Fig. 5:**
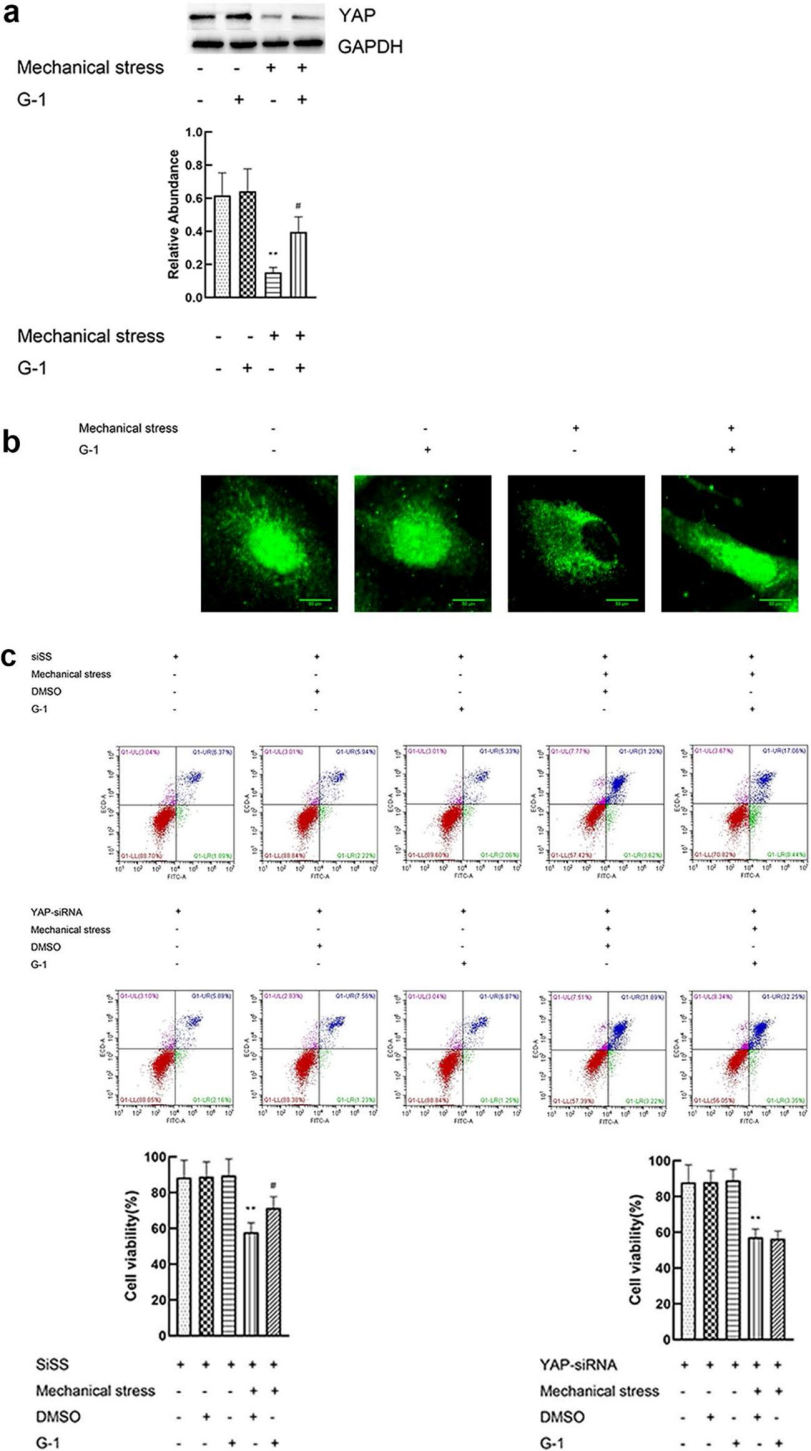

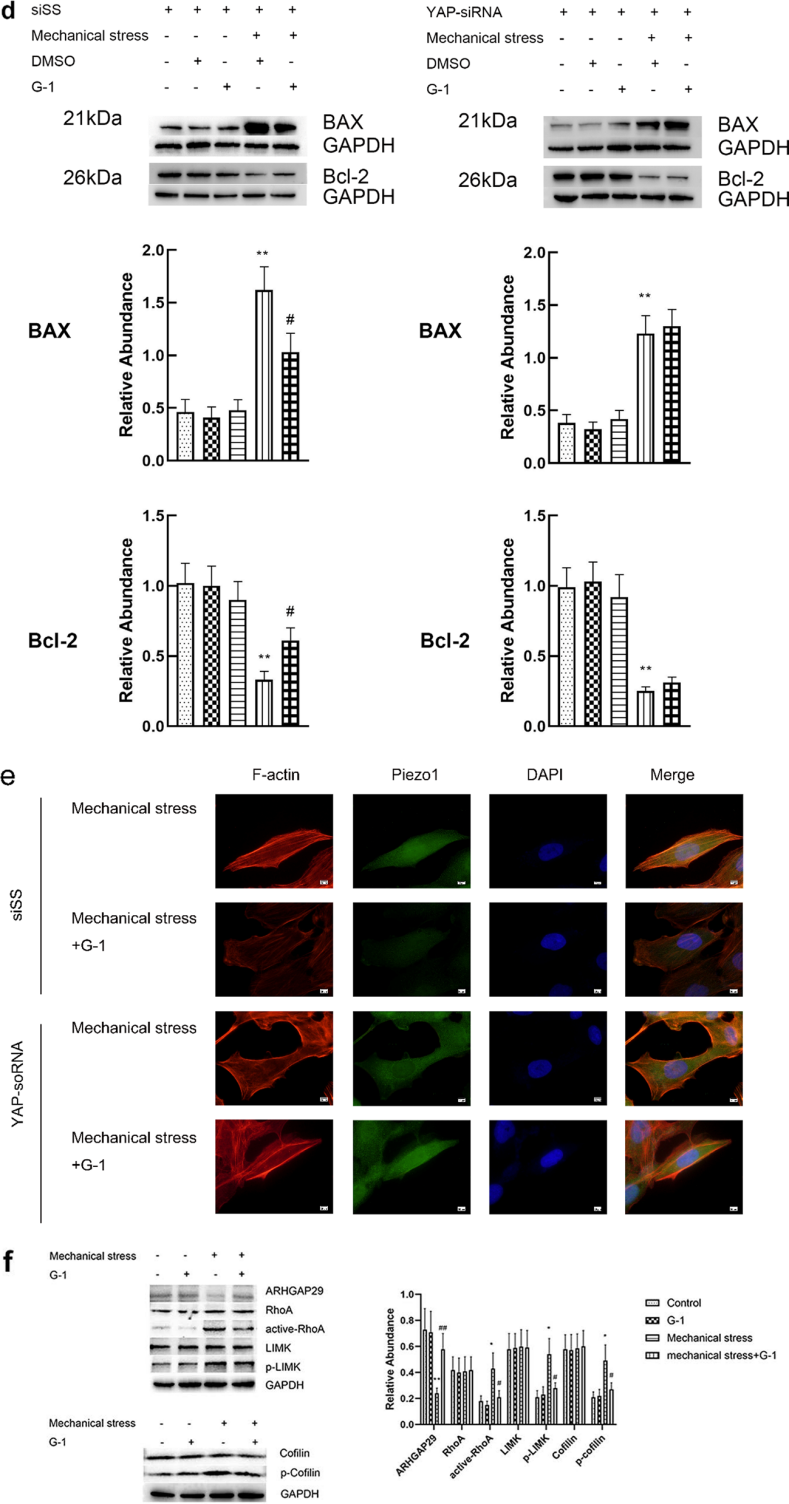
GPER regulates actin polymerization via YAP-ARHGAP29-RhoA-LIMK-Cofilin Signaling. **a** Protein expression of YAP in chondrocyte treated with G-1 or mechanical stress (n = 3), *P < 0.05, compared with control group. **P < 0.01, compared with control group. ^#^P < 0.05, compared with mechanical stress group. ^##^P < 0.01, compared with mechanical stress group. **b** Immunofluorescence staining YAP (n = 3). (c) Cell viabilities for chondrocytes with YAP or scrambled siRNA after treatment with mechanical stress, and G-1 (n = 3), *P < 0.05, compared with DMSO group. **P < 0.01, compared with DMSO group. ^#^P < 0.05, compared with mechanical stress + DMSO group. ^##^P < 0.01, compared with mechanical stress + DMSO group. **d** Protein expression of Bax and Bcl-2 for chondrocytes with YAP or scrambled siRNA after treatment with mechanical stress, and G-1 (n = 3), *P < 0.05, compared with DMSO group. **P < 0.01, compared with DMSO group. ^#^P < 0.05, compared with mechanical stress + DMSO group. ^##^P < 0.01, compared with mechanical stress + DMSO group. **e** Immunofluorescence staining F-actin and Piezo1 (n = 3). **f** Protein expression of ARHGAP29-RhoA-LIMK-Cofilin Signaling in chondrocyte treated with G-1 or mechanical stress (n = 3) *P < 0.05, compared with control group. **P < 0.01, compared with control group. ^#^P < 0.05, compared with mechanical stress group. ^##^P < 0.01, compared with mechanical stress group. All data are presented in mean ± SD

YAP, a transcription molecule involved in mechanical signaling, was also detected. G-1-mechanical stress treated chondrocyte had significantly increased YAP expression (Fig. 5a) and apparently nuclear localization (Fig. 5b) compared with mechanical stress treated cells. To confirm if YAP involved in apoptosis induced by mechanical stress, YAP was inhibited using siRNA knockdown. Compared with the scrambled siRNA, YAP-siRNA effectively blocked the anti-apoptosis effect of G-1 (Fig. 5c, d). These results suggested that YAP played a role in apoptosis regulated by G-1 and mechanical stress. In addition, we explored whether YAP has a potential regulatory effect on polymerization of actin and associated signaling pathway. The results of immunofluorescence showed that YAP silencing effectively attenuated the depolymerization of actin and inactivation of Piezo1casused by GPER activation induced by G-1 (Fig. 5e). As an important modulator of actin dynamics, ARHGAP29 reduces the activity of RhoA by promoting the transformation from GTP-bound (active) RhoA to its GDP-bound (inactive) form, and then inhibit the LIMK/cofilin pathway intimately implicated in actin dynamics. Our data showed that compared with the control group, the expression of ARHGAP29 protein in the mechanical stress group was significantly reduced, and the RhoA activity, LIMK activity and cofilin phosphorylation level were elevated, which could be rescued by G-1 treatment (Fig. 5f). Taken together, activation of GPER suppresses actin polymerization via YAP-ARHGAP29-RhoA-LIMK-Cofilin Signaling.

### G-1 activated GPER inhibits articular cartilage degeneration in vivo

**Fig. 6:**
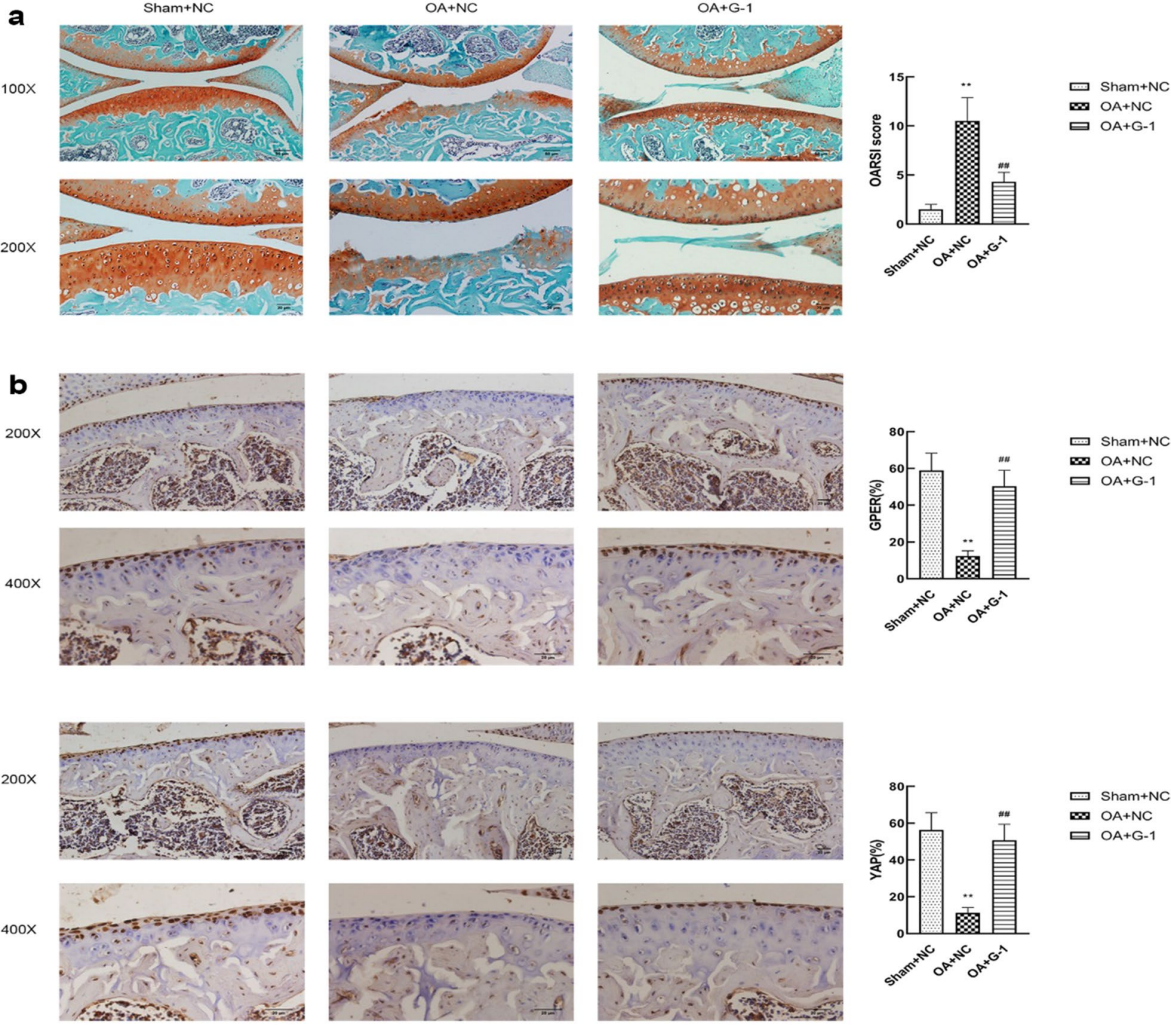
G-1 activated GPER inhibits articular cartilage degeneration in vivo. **a** Safranine and Fast Green double staining in each group and associated OARSI scores (n = 6). **b** Immunofluorescence staining GPER, and YAP (n = 6). All data are presented in mean ± SD *P < 0.05, compared with Sham + NC. **P < 0.01, compared with Sham + NC. ^#^P < 0.05, compared with OA + NC. ^##^P < 0.01, compared with OA + NC

The articular cartilages were estimated by Safranine and Fast Green double staining (Fig. 6a). The data of OARSI scores presented that the worse articular cartilage degeneration in OA + NC group than the Sham + NC group, whereas the G-1treatment attenuated this pathological process. Moreover, we used immunohistochemical staining of GPER and YAP. As shown in Fig. 6b, the G-1treatment could enhance the YAP expression in articular cartilage of rats. These data implied that intra-articular injection G-1 has a potential protective effect in vivo.

## Discussion

In this study, we found that (1) estrogen pathway is involved in the mechanical signal transduction of chondrocytes mediated by Piezo1, and the activation of GPERs rather than ERs acts a crucial role in the mechanical stress-induced chondrocyte apoptosis; (2) Expression of GPER significantly reduces in osteoarthritis cartilage and rat osteoarthritis models; (3) G-1 activated GPER to play the role of cartilage protection under mechanical stress (4) GPER activation greatly promoted the subcellular localization of transcriptional costimulatory molecule YAP from cytoplasm to nucleus (5) the nuclear localization of YAP upregulated the expression of ARHGAP29, downregulated the phosphorylation of RhoA, LIMK and cofilin, led to the depolymerization of actin (6) the depolymerization of actin blocked the stimulation of mechanical stress on Piezo1, eventually increased the viability of chondrocytes. To our best known, this is the first study to describe the comprehensive role of GPER in the chondrocyte apoptosis induced by mechanical stress via regulation of actin/Piezo1 axis by influencing YAP/ARHGAP29/RhoA/ LIMK/cofilin pathways.

Epidemiological data showed that the prevalence of OA in women after 50 years old was significantly higher than that in men and premenopausal women, and the serum estradiol level in postmenopausal OA patients was markedly lower than that in normal postmenopausal women, and the severity of knee osteoarthritis increased with the decrease of estradiol level (Zhou et al. 2018; Hussain et al. 2018; Jung et al. 2018; Gao et al. 2010). Estrogen or its modulators can effectively relieve the pain of OA patients, and slightly reduce the prevalence and the incidence of joint replacement (Xiao et al. 2016; Tsai and Liu 1992). The physiological concentration of estrogen can regulate the viability of chondrocytes and promote their proliferation and differentiation (Ge et al. 2019; Chagin et al. 2006). On the contrary, estrogen deficiency can activate the apoptosis mechanism of chondrocytes, which can make chondrocytes apoptosis, prevent the proliferation of chondrocytes, and cause the occurrence and progress of osteoarthritis (Talwar et al. 2006). It is reported that estrogen can effectively reduce the decrease of chondrocyte viability caused by mechanical stress (Imgenberg et al. 2013; Ge et al. 2019), which is consistent with the results of this study. These data demonstrated the important protective role of estrogen in articular cartilage.

Estrogen receptors can be divided into two types: conventional ER in cytoplasm and nucleus and GPER in cell membrane (Pakdel 2018). Previous researches focused on the effect of ER on cartilage, which affects the process of OA by regulating cartilage matrix metabolism and chondrocyte proliferation (Son et al. 2017). However, the role of GPER in chondrocytes is not clear. The results of microarray analysis showed that GPER expression in chondrocytes was significantly up-regulated and ER did not change significantly after Piezo1 was silenced, which was also verified in transcription and post transcription levels. The antiapoptotic effect of estrogen can be blocked by G15 or silencing GPER. In addition, G-1, can simulate the effect of estrogen to weaken the decrease of chondrocyte viability caused by mechanical stress. GPER was negatively correlated with the degeneration of cartilage in human and experimental OA, implying that GPER instead of ERs is possibly engaged in OA progression. Actually, activation of GPER significantly impacted on apoptosis of chondrocyte during OA pathogenesis, as evidenced by that G-1 was able to restrain activation of Piezo1 and cartilage degeneration due to surgery-induced OA. Furthermore, we revealed the potential molecular mechanism of GPER for OA cartilage protection in vitro and in vivo. In general, GPER strictly controls OA progression via critically regulating the actin dynamics, prerequisites for Piezo1 activation, regulated by mechanical stress. Therefore, we elucidate GPER as a crucial mechanoregulator of cartilage and its activation accelerates the progression of OA.

It’s common knowledge that the transcription of ARHGAP29 are promoted by YAP, and consequently inhibit the RhoA/LIMK/cofilin pathways, eventually resulting in actin depolymerization (Qiao et al. 2017). However, the role of YAP in articular cartilage is controversial. It is reported that YAP expression is up-regulated in human and mouse osteoarthritis cartilage and chondrocytes, and silencing of YAP can inhibit IL-1β induced chondrocyte apoptosis (Gong et al. 2019). Yap knockout exerted maintaining the expression of type II collagen and inhibiting cartilage degeneration in the OA model (Ying et al. 2018). In addition, intra-articular injection of YAP-siRNA or verteporfin, a selective inhibitor of YAP, significantly reduced abnormal subchondral bone formation, and preserved cartilage homeostasis in mice OA model (Delve et al. 2020). On the contrary, some scholars think that overexpression of YAP is helpful to preserve the integrity of articular cartilage, and the absence of YAP leads to the destruction of cartilage. YAP suppresses NF-κB pathway by inhibiting IKKα/β activation to reduce intra-articular inflammation. Intra articular injection of YAP markedly decreased the occurrence of OA in mice (Deng et al. 2018). This study elucidated that the expression of YAP was elevated and subcellular localization was transferred from cytoplasm to nucleus after GPER activation, causing downregulation of mechanical stress-mediated signaling and polymerization of actin. In addition, we observed the decrease of YAP expression in rat OA model. After YAP silencing, G-1 treatment of chondrocytes still could not improve the decrease of chondrocyte viability induced by Piezo1 mediated mechanical stress.

Mechanical stress is an important factor of apoptosis of chondrocyte during OA (Zhao 2020). Therefore, suppression of mechanical stress signaling is crucial to alleviate apoptosis of chondrocyte for the prevention of cartilage degeneration (Lee et al. 2017b). Our previous study found that Piezo1 mediates the apoptosis of chondrocytes induced by mechanical stress (Li et al. 2017). Our study shows that GPER interacts with Piezo1 to participate in mechanical stress-mediated signal transduction and the pathogenesis of OA. In addition, previous studies have reported that GPER can also interact with other membrane proteins (Zhang et al. 2018). Microglia GPER acts as a neuroprotective role by inhibiting microglial inflammation mediated by TLR4. Several potential pathways may be involved in GPER-induced down-regulation of TLR4 in microglia. First, GPER may indirectly regulate TLR4 transcriptional activity through activation of a second messenger signaling cascade, such as adenylate cyclase-cAMP signaling (Alexander et al. 2017), PI3-kinase/Akt (PI3K/AKT)-dependent signaling and Src protein kinase-epidermal growth factor receptor pathway (Alexander et al. 2017; Prossnitz and Barton 2011; Filardo and Thomas 2012). In addition, GPER upregulates BDNF by regulating PI3K/Akt and MAPK/ERK signaling pathways. Second, GPER may inhibit TLR4 expression through the "bridge molecule", namely the RAB protein (Wang et al. 2007). Rab proteins are involved in TLR4-mediated inflammation. RAB8A and RAB10 reduce TLR4 expression in the membrane and its downstream pro-inflammatory cytokines (Wang et al. 2010; Luo et al. 2014), and RAB7B reduces TLR4 expression in macrophages (Wang et al. 2007). Combined with literature reports, we speculate that GPER interacts with piezo1 through YAP/ARHGAP29/RhoA/ LIMK/cofilin pathway, and may also interact with other signaling pathways (PI3K/Akt, etc.) and molecules (RAB protein) to regulate mechanical signal transduction in chondrocytes, which needs further experiments to prove.

## Conclusions

All in all, we propose a novel regulating and controlling mechanism by uncovering that GPER is an critical modulator of mechanical stress-mediated activation of Piezo1 and trigger of apoptosis of chondrocyte mechanically via the YAP/ARHGAP29/RhoA/ LIMK/cofilin pathway during OA progression, and elucidate the underlying application value of GPER as cure targets for OA.

## Supplementary Information

**Additional file 1.** Supplementary material.

## Data Availability

The data that support the findings of this study are available from the corresponding author upon reasonable request.
