# Supplementary material for: G protein coupled estrogen receptor attenuates mechanical stress-mediated apoptosis of chondrocyte in osteoarthritis via suppression of Piezo1
Source: Mol Med. 2021 Aug 28;27:96. doi: 10.1186/s10020-021-00360-w (PMC8403401; doi:10.1186/s10020-021-00360-w)
Supplement: Supplementary file 1 — Additional file 1. Supplementary material. [file 10020_2021_360_MOESM1_ESM.docx]

**Additional Data**


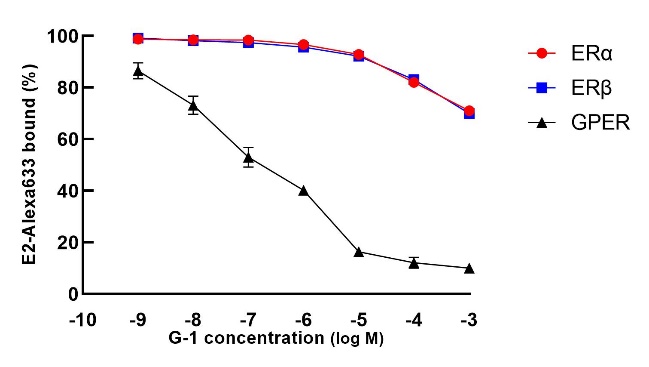

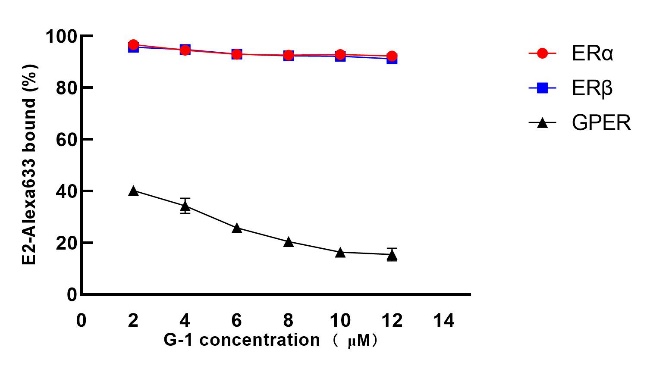


Additional file 1: Dose-response curve of G-1. GPER-siRNA, ERα-siRNA and ERβ-siRNA were used to knockdown the corresponding expression in OA chondrocytes. Fluorescently labeled estrogen was added to bind to GPER, ERα and ERβ and then indicated concentration of G-1 was used to competitively bind to GPER, ERα and ERβ. The change of fluorescence was detected and Prism 8.0 (Graph Pad) was used to analyze the competitive binding data. The binding of G-1 to GPER increased gradually with the increase of dose. At the concentration of 10 µM, G-1 had a higher ratio of GPER activation and a weaker effect on both ERα and ERβ (n=3).


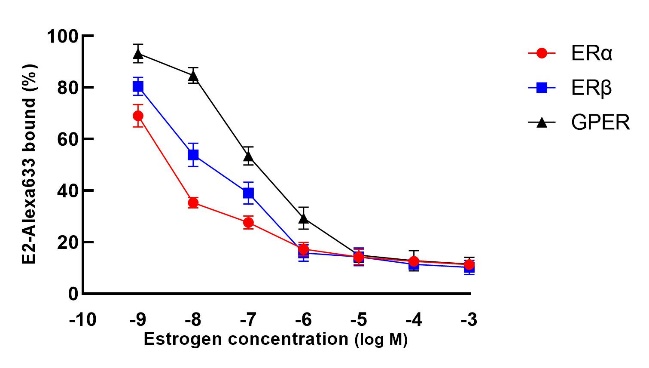

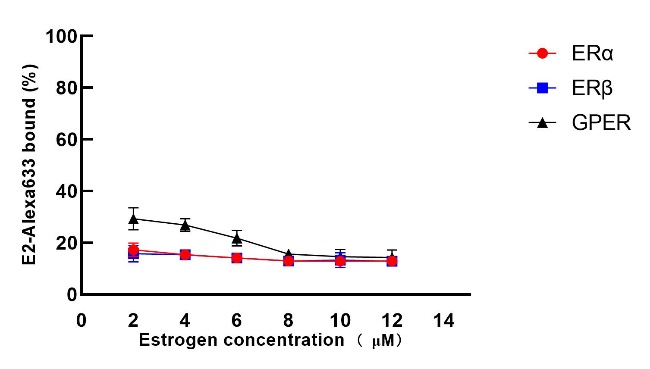


Additional file 2: Dose-response curve of estrogen. GPER-siRNA, ERα-siRNA and ERβ-siRNA were used to knockdown the corresponding expression in OA chondrocytes. Fluorescently labeled estrogen was added to bind to GPER, ERα and ERβ and then indicated concentration of estrogen was used to competitively bind to GPER, ERα and ERβ. The change of fluorescence was detected and Prism 8.0 (Graph Pad) was used to analyze the competitive binding data. The binding of estrogen to GPER increased gradually with the increase of dose. At the concentration of 8 µM, estrogen had a higher ratio of GPER, ERα and ERβ activation (n=3).
